# Complete genome sequence analysis of *Sarbecovirus* (severe acute respiratory syndrome-related coronaviruses) from Zimbabwean insectivorous bats

**DOI:** 10.1186/s12864-025-12015-9

**Published:** 2025-09-26

**Authors:** Vimbiso Chidoti, Anaïs Loisier, Taona Zinyakasa, Mathieu Bourgarel, Valérie Pinarello, Gift Matope, Ngoni Chiweshe, Dorothée Missé, Ellen Mwandiringana, Hélène De Nys, Florian Liégeois

**Affiliations:** 1 Biotechnology Centre, Faculty of Veterinary Science, University of Zimbabwe, Harare, Zimbabwe; 2 MIVEGEC, IRD, CNRS, Université de Montpellier, Montpellier, France; 3 ASTRE, CIRAD, INRAE, Université de Montpellier, Montpellier, France; 4 Centro de Biotecnologia, Universidade Eduardo Mondlane, Maputo, Mozambique; 5 UMR ASTRE, CIRAD, Harare, Zimbabwe; 6 Faculty of Veterinary Science, University of Zimbabwe, Harare, Zimbabwe

**Keywords:** Bat sarbecoviruses, *Nycteris macrotis*, *Rhinolophus simulator*, Full genome, Zimbabwe

## Abstract

Bat-borne severe acute respiratory syndrome-related coronaviruses (bat SARSr-CoVs), belonging to the *Sarbecovirus* subgenus of the *Betacoronavirus* genus pose a potential risk for zoonotic spillover. This study reports the complete genome sequences of bat sarbecoviruses from *Nycteris macrotis* and *Rhinolophus simulator* insectivorous bat species in Zimbabwe. Using genome walking and nested PCR approaches, six full viral genomes were amplified, sequenced and assembled: one from *Nycteris macrotis* (MAG1042) and five from *Rhinolophus simulator* bats species (MAG388, MAG562, MAG575, MAG850, and MAG859). Comparative genomic analysis revealed a high degree of sequence conservation (99.6–99.9%) among the *Rhinolophus simulator*-characterised sequences, while the *Nycteris macrotis* characterised sequence exhibited 96.7% identity with the *Rhinolophus simulator* consensus (MAG Cons) genomes, suggesting genus-specific evolution. Furthermore, the spike region exhibited notable divergence, with only 88% sequence identity between the two genera. A complete absence of ORF8, and truncation of ORF7b genes were observed, while the *RNA dependent RNA polymerase* genes were highly conserved across all strains. Phylogenetic analysis demonstrated distinct continental clustering, with the Zimbabwean genomic sequences forming a well-supported clade within the African lineage (Lineage 4). These newly characterized Zimbabwean sarbecoviruses sequences represent the most recent evolutionary branches within the Lineage 4-African clade, indicating ongoing viral diversification in local bat populations. These findings expand understanding of SARSr-CoV diversity in African bats and underscore the importance of geographical isolation in viral evolution.

**Supplementary Information:**

The online version contains supplementary material available at 10.1186/s12864-025-12015-9.

## Introduction

Coronaviruses (CoVs) are a family of viruses that infect a wide diversity of animals, including bats, which serve as natural reservoirs for several potential zoonotic CoVs [1–4]. These viruses are characterized by a high frequency of recombination due to their unique viral replication mechanism [2, 3, 5]. Recombination, together with genetic changes, insertions, substitutions and deletions, play an important evolutionary role in CoVs genome, contributing to the emergence of novel strains [2].

In 2005, a novel *Coronavirus* related to human SARS-CoV-1 was discovered in *Rhinolophus* bat species in China and was subsequently termed bat SARS-related coronaviruses (bat SARSr-CoVs) belonging to the subgenus *Sarbecovirus* [6–10]. This discovery sparked widespread interest in bat sarbecoviruses due to their potential role in the emergence of severe acute respiratory syndrome (SARS-CoV-1) in 2003 and the recent SARS-CoV-2 pandemic [7]. Bat sarbecoviruses share a similar genome organization with human SARS-CoVs, showing 88–96% genetic identity [2, 11–14]. In particular, Rehman et al. and Kim et al. reported bat sarbecoviruses in China with a genetic identity of 92% with the human SARS-CoV-1 Tor2 strain [13, 14]. In contrast, Zhou et al. (2020) identified a 96% genetic identity between SARS-CoV-2 and a previously detected bat sarbecovirus strain [4]. Additionally, Geng and Zhou, reported that these bat sarbecovirus genomes exhibited 88–92% similarity with human SARS-CoV-1 [2], highlighting the genetic diversity among bat coronaviruses and their varying degrees of relatednes to human strains.

Further genetic analysis has confirmed the high level of diversity among bat sarbecoviruses, suggesting that these viruses are widespread in various bat species across the globe [8, 9, 12] This diversity is shaped not only by long-term host-virus co-evolution but also by frequent recombination events, especially in the spike gene regions involved in host cell entry [15]. Studies in natural hosts initially discovered bat sarbecoviruses in *Rhinolophidae* (horseshoe) bats in China and further research has increasingly supported the notion that bats from this family are key reservoirs of sarbecoviruses [3, 16–18]. *Hipposideros* bats have also been identified alongside *Rhinolophidae,* as part of the main bat families that carry sarbecoviruses [2, 8, 9]. Additionally, evidence of genetic introgression within *Rhinolophidae* suggests frequent interspecies contact, potentially promoting viral exchange and host switching opportunities [15]. However, despite these discoveries, no definitive conclusion has been reached regarding whether bat sarbecoviruses are the direct ancestors of human SARS-CoVs [2–4, 12].

In Africa particularly in Kenya, Rwanda and Uganda, full genomes of bat sarbecoviruses have been generated from viruses detected in *Rhinolophus* bats. These findings were consistent with similar studies conducted in Europe and Asia [15, 19–21]. However, genomic data on bat betacoronaviruses (β-CoVs) in African countries remains scarce beyond these few studies in East Africa. Furthermore, in their review Markotter et al., (2020) indicated that surveillance of bat coronaviruses in Africa largely relied on partial *RNA-dependent RNA polymerase* (*RdRp*) gene sequences, with limited full-genome data available due to challenges such as low viral loads, insufficient sample material, and limited access to high-throughput sequencing technologies [22]. The majority of studies were geographically concentrated in only a few countries and focused on partial genomic data, which restricts our understanding of the full genetic diversity, evolutionary dynamics, and zoonotic potential of bat CoVs across the continent.

Our previous study (Chidoti et al2022) identified bat sarbecoviruses in insectivorous bats from Magweto Cave, Zimbabwe, through partial sequencing of the *RNA-dependent RNA polymerase* (*RdRp*) gene [23]. Using consensus PCR and Sanger sequencing, we classified these viruses into betacoronavirus (β-CoV) subclades and designated the sequences as B-SVG-06 and B-SVG-07 (B = Beta, SVG = Sub Viral Group) based on their phylogenetic clustering [23]. While this work confirmed the circulation of bat sarbecoviruses in Zimbabwean bat populations, it did not include full genome sequencing or detailed genomic characterization.

This study builds upon these previous findings by providing the first full genome sequences of six sarbecoviruses (MAG- 388, 562, 575, 850, 859 and 1042) characterised from insectivorous bats in Zimbabwe. Given the increasing recognition of bat-associated CoVs as potential zoonotic threats, this work aims to characterize the genetic diversity of these viruses and examine their genomic and phylogenetic relationships with both human SARS-CoVs and other bat sarbecoviruses from Africa and globally.

## Methodology

### Study sites

**Fig. 1:**
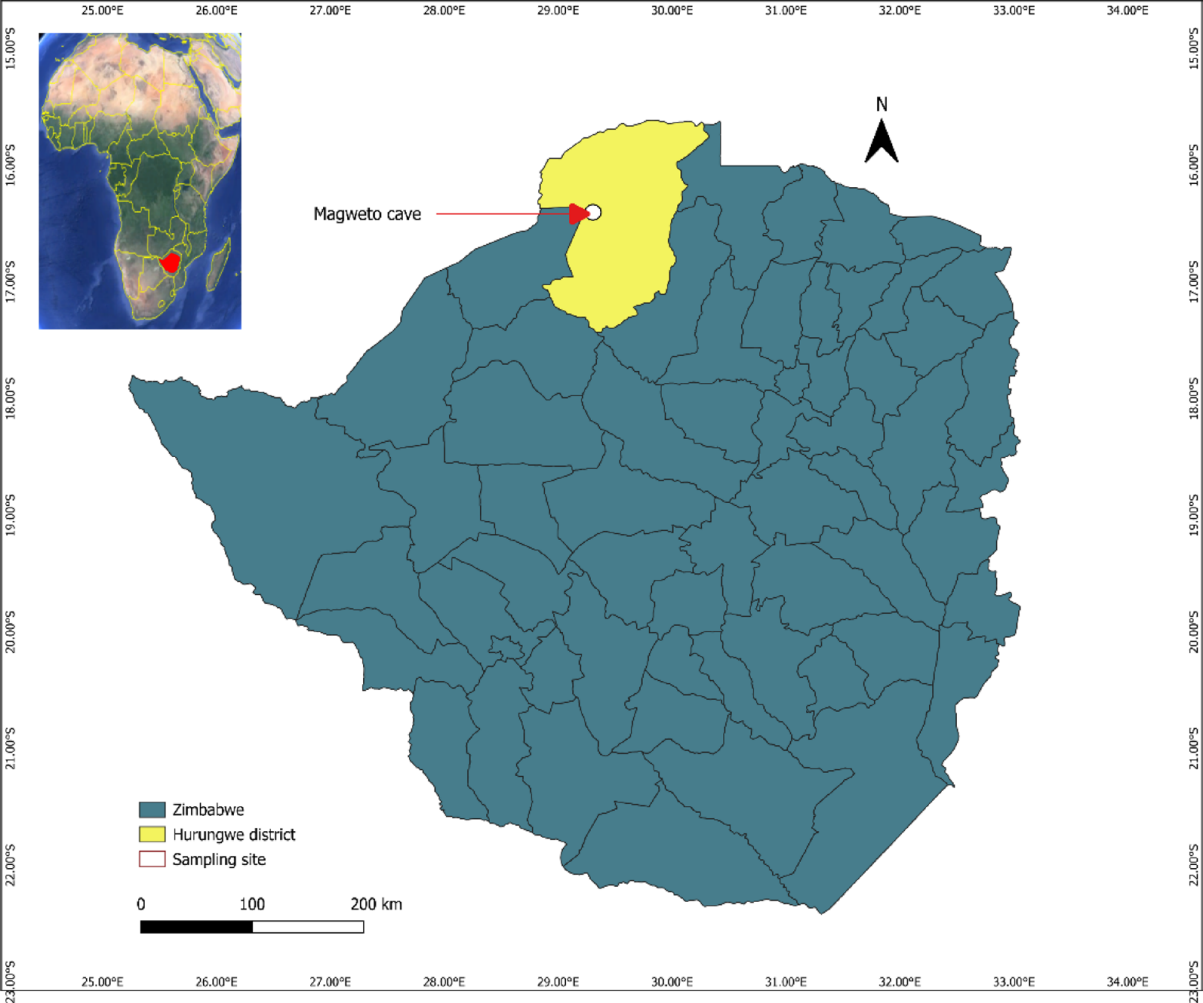
The sampling site of insectivorous bats in Zimbabwe

This study was conducted at Magweto Cave, Zimbabwe (17°06′38 “S, 29°11′54 “E) (Fig. 1), a known roosting site for colonies of insectivorous bats from the *Hipposideridae*, *Rhinolophidae*, *Nycteridae*, *Miniopteridae*, and *Macronycteridae* families. The cave is utilized by the surrounding community for various activities, including cultural and religious ceremonies and guano collection. On several occasions, bat hunters were encountered inside the cave, and they confirmed hunting and consuming bats, highlighting potential zoonotic transmission risks [23].

### Bat faeces sampling

Fecal samples used in this study were collected at Magweto Cave, Zimbabwe, during multiple sampling events between August 2020 and July 2021. Sampling was performed non-invasively by placing collection sheets beneath bat roosting sites to gather freshly dropped feces. An area of 20 cm × 20 cm was considered representative of a bat defecation area, from which a single and fresh fecal pellet was collected and preserved in a homemade nucleic acid preservative solution (http://www.protocol-online.org/prot/Protocols/RNAlater-3999.html) for laboratory analysis. The analysed samples were collected on the following collection dates: MAG-388 on 20/10/2020, MAG-562 and MAG-575 on 22/10/2020, MAG-850 and MAG-859 on 04/03/2021, and MAG-1042 on 06/03/2021.

### Viral detection and RNA/DNA extraction

Both RNA and DNA were extracted from 200 µL of fecal samples using the 5X MagMax Pathogen RNA/DNA Kit (Thermo Fisher Scientific, Illkrich, France), following the manufacturer’s protocol. The samples, preserved in a homemade nucleic acid preservative solution, were processed as described by Chidoti et *al*. (2022) [23]. The extracted RNA/DNA was subjected to reverse transcription polymerase chain reaction (RT-PCR) using random hexamers, oligoDT (Thermo Fisher Scientific, Illkrich, France) and pan-generic coronavirus primers to amplify a 440 bp of CoV *RdPd* gene according to Chu et al. [24]. Negative and positive controls were used to validate the assays and ensure that no contamination occurred during processing.

### Initial detection of Viral RNA and phylogenetic analysis

Following PCR amplification, the 440 bp PCR product was purified and Sanger sequenced (LGC, Berlin, Germany). Following sequencing, the generated sequences were compared to a database of sequences online using Basic Local Alignment Search Tool (BLAST) (https://blast.ncbi.nlm.nih.gov/Blast.cgi) to identify the amplified sequence. Phylogenetic analysis was conducted on all identified partial coronavirus sequences, utilizing a diverse collection of partial coronavirus sequences sourced from GenBank. The dataset represented various continents and hosts, encompassing coronaviruses from humans, bats, and other mammalian species. Maximum-Likelihood (ML) phylogenies were inferred using IQ-TREE v1.6.12 with 1000 bootstrap replicates to evaluate the evolutionary relationships of the sequences. Six sequences, five from *Rhinolophidae* and one from *Nycteridae* bat families clustered closely with known human SARS-CoVs and bat sarbecoviruses [23]. The six samples analyzed in this study (MAG388, MAG562, MAG575, MAG850, MAG859, and MAG1042) correspond to those previously grouped as B-SVG-06 and B-SVG-07 in Chidoti et al. (2022) [23], where they were identified based on partial *RdRp* sequences.

### Full genome amplification and sequencing

**Reverse transcription:** RNA was reverse transcribed into cDNA using random hexamers and Oligo dT primers. A 5 µL RNA template was incubated in a 13 µL reaction with 1 µL random hexamers, 0.5 µL Oligo dT, 0.4 µL 10 mM dNTPs, and 5.5 µL molecular-grade water at 65 °C for 5 min. Then, 4 µL 5 × Buffer, 2 µL 0.1 M DTT, and 1 µL RNAse OUT were added, followed by incubation at 37 °C for 2 min. M-MLV reverse transcriptase (200U) was then added, and the reaction proceeded at 25 °C for 10 min, 37 °C for 50 min, and 70 °C for 15 min. The cDNA was stored at − 20 °C.

### Genome amplification

Full genomes were obtained using genome-walking approach with nested and semi-nested PCR to amplify CoV DNA fragments from one *Nycteris macrotis* (MAG1042) and five *Rhinolophus simulator* bat samples (MAG388, MAG562, MAG575, MAG850, MAG859). Firstly, initial degenerate primers were designed from human SARS-CoVs and bat sarbecoviruses alignments in order to amplify different fragments across the genome (Additional file 1, Additional file 2). Based on these initial fragments amplified, specific or slightly degenerate primers were designed to cover the full genomes (Additional file 1, Additional file 2). PCR reactions (50 µL) contained 5 µL cDNA template, 5 µL DreamTaq Buffer (10X), 0.5 µL (2,5 U) DreamTaq polymerase (Thermo Fisher Scientific, Illkrich, France), 5 µL Buffer, 0.4 µL (25 Mm) dNTPs (Thermo Fisher Scientific, Illkrich, France), and 20 pmol of each primer and molecular grade water. The following amplification procedures were applied: 95 °C for 2 min (Denaturation); 40 cycles of 95 °C for 20 s (denaturation), 50 °C for 30 s, 70 °C from 1 min to 4.5 min according to the amplicon size expected (elongation). The visualization of PCR products was conducted using gel electrophoresis of 1.5% agarose gel stained with 0.1% ethidium bromide at 80 V for 40 min.

### Bat species identification

The species identification of the six bat samples analyzed in this study was previously performed and described in Chidoti et al. (2022) [23]. In that study, five of the six samples were genotyped to the species level using cytochrome b PCR, while one sample (MAG-388) was identified using 12S rRNA PCR. The detailed protocols for molecular identification are available in the initial publication.

### Sanger sequencing and sequence cleaning

Amplified CoV amplicons from *Nycteris* and *Rhinolophus* species were Sanger sequenced (LGC, Berlin, Germany) using both second-round PCR primers and sequencing-specific primers. Sequence data were processed in Geneious Prime (version 2023.2) [25], including quality-trimming to remove low-quality base calls and primer sequences, followed by alignment and assembly into contigs. Final consensus sequences were generated by resolving sequence discrepancies through majority-rule consensus.

Nucleotide sequence identities between the Zimbabwean sequences (*Nycteris*-MAG1042 and the consensus sequence from *Rhinolophus* samples MAG388, MAG562, MAG575, MAG850, and MAG859) were compared to other African and Asian SARS-related coronaviruses and SARS-CoV-2 using multiple sequence alignment in Geneious v2024 to assess genetic diversity and conservation.

### Full genome phylogenetic analysis

The assembled sequences were aligned using MAFFT v7.490 [26] against full-genome reference sequences of bat *Sarbecovirus* from Africa (Kenya, Rwanda, Uganda) [19, 20], Europe (Russia, Bulgaria) [5, 27], and Asia. To ensure alignment accuracy, the sequences were further verified using MEGA v.11 [28], which allowed us to detect and correct any potential ambiguities. Maximum-Likelihood (ML) phylogenies were then constructed using IQ-TREE v1.6.12 with 1000 bootstrap repetitions and the GTR + I + G substitution model to assess the robustness of the tree and evaluate the evolutionary relationships of the sequences [29]. Bootstrap support values were computed using the UFBoot2 (Ultrafast Bootstrap) approximation method [30], the default for IQ-TREE ML analysis, which provides a reliable and efficient alternative to traditional bootstrap approaches. analysis provided comprehensive insights into the evolutionary history, genetic diversity, and potential zoonotic transmission pathways of SARS-related CoVs found in Zimbabwean bats.

### Full genome genetic identities

The genetic identities of Zimbabwean full genome sequences (*Nycteri*s-MAG1042, *Rhinolophus*-MAG388, -MAG562, -MAG575, -MAG850, and -MAG859) were determined by comparing them with African, European, and Asian bat sarbecoviruses and also human SARS-CoVs using the Pairwise Identity function in Geneious Prime (version 2023.2) software [25]. After aligning the sequences with reference genomes using MAFFT v7.490 [26], the aligned full genomes were analyzed using the Statistics → Pairwise Identity option within the software. Gaps in the sequence alignment were treated as differences to account for insertions and deletions that may hold evolutionary significance. The resulting pairwise identity matrix quantified the genetic similarities between the Zimbabwean bat sarbecoviruses genomic sequences and reference sequence, with each value representing the percentage of identical nucleotide positions between two sequences. This pairwise identity approach enabled precise quantification of sequence conservation across the viral genomes, facilitating the identification of closely related viral strains and potential recombination events. The final output, a pairwise identity matrix, visually represented the genetic similarity between the sequences, with percentage values indicating the degree of sequence identity.

### Similarity plot analysis

A bootscan analysis, utilizing a sliding window approach, was conducted to identify potential recombination events between the Zimbabwean genomic sequences: *Nycteris macrotis* MAG1042 and MAG-Cons (MAG388, MAG562, MAG575, MAG850, and MAG859) from five different *Rhinolophus simulator* bat species, as well as African and European bat sarbecoviruses. The analysis was performed using SimPlot version 3.5.1 [31] applying a 500 bp sliding window with a step size of 50 bp.

Based on the SimPlot results, separate phylogenetic analyses were conducted for genomic regions labeled A to G in the SimPlot graph, representing areas of observed similarities and dissimilarities across the genome. These regions were defined according to potential recombination break point as well as key functional and structural genome elements: Regions A, B, and C correspond to ORF1ab (nsp1–nsp16); Regions D, E, and F correspond to the Spike (S) gene; and Region G comprises ORF3a, Envelope (E), and Membrane (M) genes. Additionally, Region E covers ORF6 to ORF8 and the Nucleocapsid (N) gene. These analyses were performed as described in the full genome analysis section.

## Results

### Host species identification

The host species were identified through Cytochrome b or 12S mitochondrial gene, five of the six sequences had their genotyping identified to the species level whereas for MAG-388 it could be identified to genus level by 12S PCR (Table 1). 

**Table 1:**
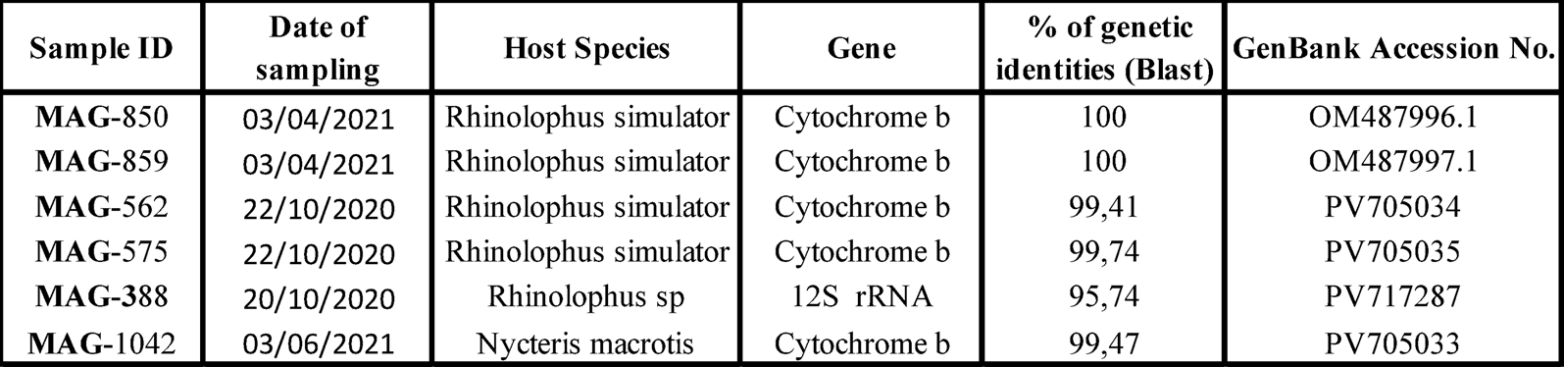
Host species identification and corresponding CYB or 12 S RNA GenBank accession numbers for the six Bat described in this study

### Genomic structure

The complete genomes of six Zimbabwean bat *Sarbecovirus* ranged from 29,102 to 29,181 nucleotides in length, displaying the typical *Sarbecovirus* genome structure: ORF1ab, structural genes (S, E, M, N), and accessory ORFs (6, 7a, 7b, and 10). All genomes lacked ORF8 and exhibited a truncated ORF7b, missing 24 nucleotides at the 3′ end—features consistent with other African and European bat sarbecoviruses. Additionally, ORF10 was truncated in all sequences. The spike (S) gene lacked the ACE2-binding motif, suggesting divergence in receptor usage and potential evolutionary adaptation to local bat hosts (Table 2).

**Table 2:**
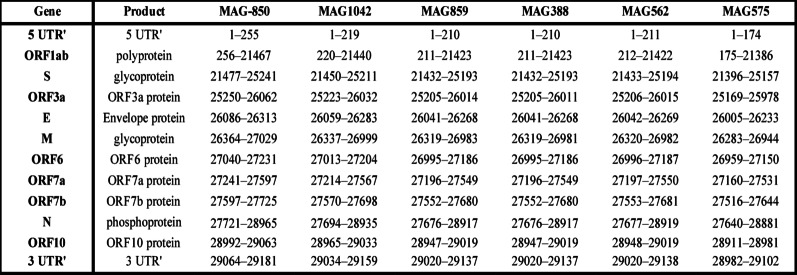
The table shows the nucleotide coordinates of annotated genes and their corresponding protein products from six bat sarbecoviruses genomes (MAG-850, MAG1042, MAG859, MAG388, MAG562, MAG575)

Coordinates are based on Sanger-sequenced genomes from Zimbabwean insectivorous Bats. Gene names correspond to standard coronavirus genomic nomenclature based on orfs. Partial regions (5’ UTR and 3’ UTR) are also included when available

### Phylogenetic analyses

**Fig. 2:**
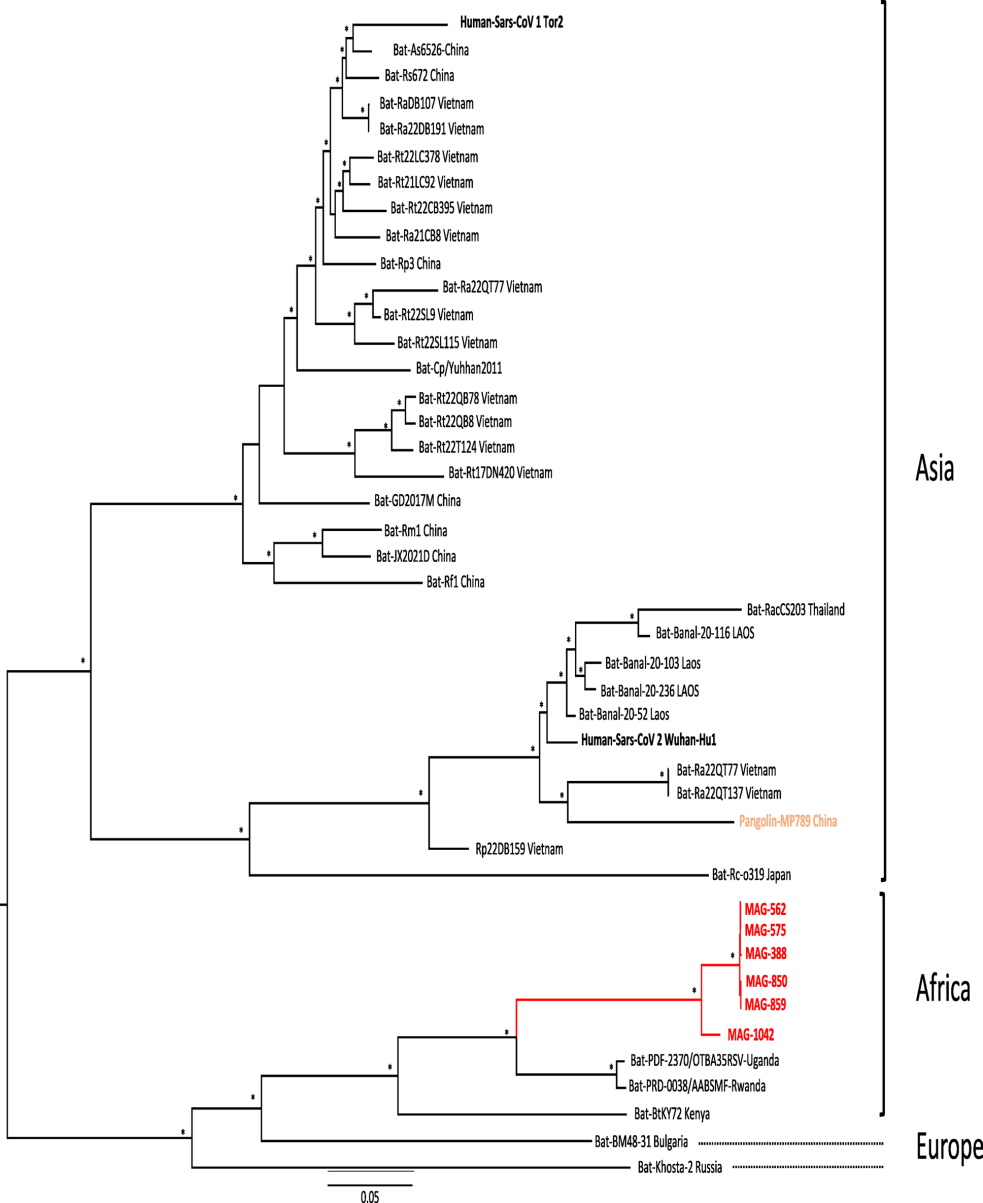
Phylogenetic analysis of full bat sarbecoviruses genomes characterised in Zimbabwean insectivorous bat samples. Zimbabwean bat sarbecoviruses full genomes are shown in red, Human SARS-CoVs in bold and pangolin sarbecoviruses in orange. Maximum-Likelihood (ML) phylogeny was constructed using IQ-TREE v1.6.12 using the UFBoot2, with 1000 bootstrap repetitions and the GTR + I + G substitution model. The asterisks represent the bootstrap support ≥ 70%. Bar scale represented the number of NA substitution per site. The tree was annotated and visualised using FigTree v1.4.4. References used in this analyse are detailed in Additional file 3

Phylogenetic analysis of full genome sequences from Zimbabwe, alongside reference strains from Africa, Europe, and Asia, revealed substantial genetic diversity. The phylogenetic tree exhibits distinct geographical clustering, highlighting regional evolutionary patterns (Fig. 2).

Asian strains displayed high genetic diversity, forming multiple sub-clusters encompassing well-documented bat sarbecoviruses from China, Vietnam, Laos, and other countries, reflecting their diverse evolutionary histories [12, 32]. African strains, including Zimbabwean sequences (MAG388, MAG562, MAG575, MAG850, MAG859, and MAG1042), formed a distinct phylogeographic clade (Fig. 2). Within this African clade, sequences from Uganda and Rwanda exhibited similar evolutionary patterns, whereas the Kenyan strain represented an earlier divergence. The Zimbabwean sequences, clustering most recently within this clade, suggest ongoing adaptation or diversification, with their close genetic similarity indicating a conserved genomic structure and a potential unique sub-lineage of *Sarbecovirus* in local bat populations (Fig. 2).

Overall, the phylogenetic tree underscores strong geographical evolutionary relationships among sequences from the same continent (Fig. 2). Zimbabwean sequences clustered closely with other African strains, while European sequences formed a distinct clade, separate from both African and Asian groups.

### Genetic identities of Zimbabwean bat sarbecoviruses

Whole-genome identities ranged from the mid-70% to over 90%, with African strains from Uganda, Rwanda, and Kenya exhibiting high conservation, suggesting close genetic relationships and low divergence within the region (Table 3). Furthermore, the *Rhinolophus simulator* bat sequences MAG Cons: MAG388, MAG562, MAG575, MAG850, and MAG859 displayed remarkable genetic conservation, sharing 99.6% identity among themselves. In contrast, MAG1042, derived from *Nycteris macrotis*, exhibited 96.7% identity with the *Rhinolophus simulator* MAG Cons sequences, indicating genetic divergence between these bat genera. Notably, MAG1042 shared only 88% identity to *Rhinolophus simulator* consensus in the Spike (S) region, suggesting ongoing evolutionary changes in this genomic region, potentially driven by adaptation to different hosts (Table 3).

**Table 3:**
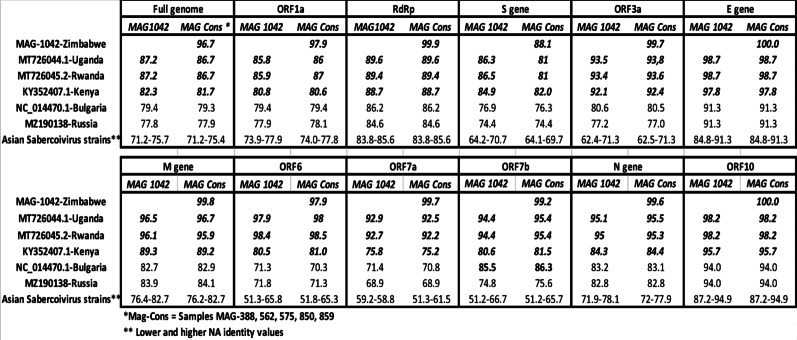
Percentage of nucleotide identity of *Nycteris macrotis* MAG1042 CoV genome from Zimbabwe in comparison with sequences from Zimbabwean *Rhinolophus simulator* MAG cons encompassing MAG388, MAG562, MAG575, MAG850, and MAG859 together with reference sequences from GenBank Bat sarbecoviruses described in other African countries and also Asia, and Europe

African sequences are represented in bolditalic. Only the lowest and highest percentage values from Asian strains are shown. Reference sequences accession numbers used for this study are provided in (Additional file 3)

### SimPlot recombinant analysis of Zimbabwean bat sarbecoviruses

**Fig. 3:**
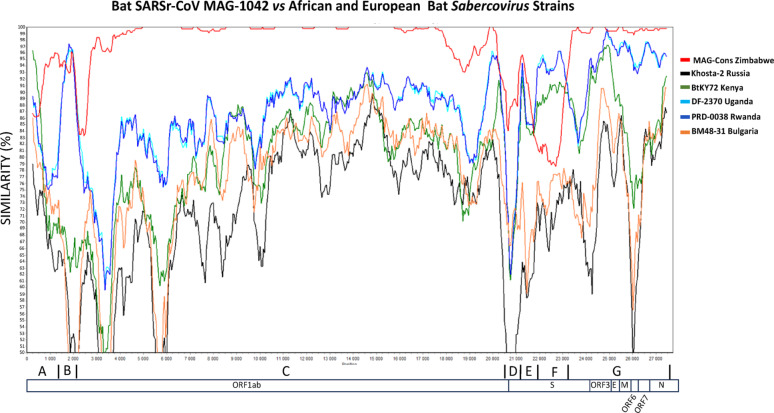
SimPlot analysis comparing the MAG-1042 genomic sequence with other Zimbabwean bat sarbecoviruses (MAG Cons: − 388, MAG-562, MAG-575, MAG-850, and MAG-859) genomes, as well as selected African (Rwanda_PRD-0038, Uganda_DF-2370 and Kenya_BtKY72) and European bat sarbecoviruses. The analysis was performed using SimPlot version 3.5.1, applying a 500 bp sliding window with a 50 bp step size

SimPlot analysis of Zimbabwean bat sarbecoviruses with reference sequences revealed distinct evolutionary patterns. Comparisons with bat sarbecoviruses from Africa, Europe, and Asia, including SARS-CoV-2 displayed variable genetic similarities and suggested potential recombination events (Fig. 3). Analysis identified conserved regions in the ORF1ab and N genes across all bat sarbecoviruses (Fig. 3). These conserved segments include essential viral proteins, such as the *RNA-dependent RNA polymerase* (*RdRp*) which is encoded by ORF1a and ORF1b. However, MAG1042 exhibited significantly lower similarity to SARS-CoV-2 and the European bat sarbecoviruses in the ORF3a and Spike (S) genes (Fig. 3). The Zimbabwean strains (represented in red) clustered closely with other African strains from Kenya, Uganda, and Rwanda. Regions of fluctuating similarity among the strains pointed to possible recombination events. While MAG-1042 shares the highest overall nucleotide identity with the MAG Cons genome, all from Zimbabwe, specific regions particularly region B and parts of the spike gene exhibit higher similarity with the Rwanda and Uganda bat sarbecoviruses, suggesting possible recombination or shared ancestry in these regions.

**Fig. 4:**
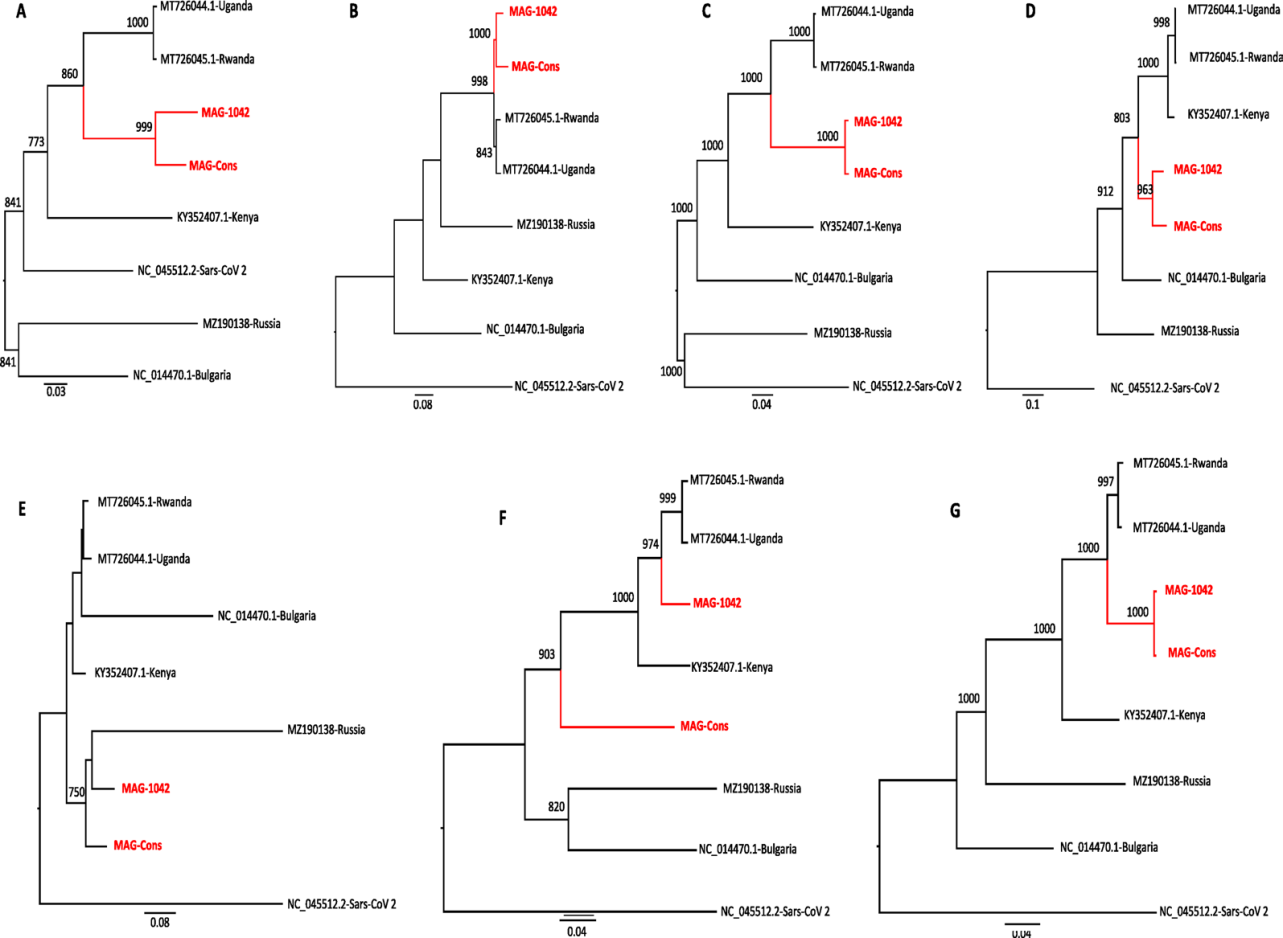
Phylogenetic analysis of MAG-1042 and MAG Cons, based on SimPlot regions (**A**, **B**, **C**, **D**, **E**, **F**, and **G**) from full genome sequences. Reference sequences are shown in black, while Zimbabwean bat sequences are highlighted in red. MAG Cons comprises of all sequences from *Rhinolophus simulator* (n = 5) and MAG-1042 from a *Nycteris macrotis* bat. Only bootstrap values ≥ 70% are shown. Bar scale represented the number of NA substitution per site

Regions A to G in the SimPlot analysis (Fig. 3) exhibited significant variation, indicating potential recombination events or areas of adaptive evolution in sarbecoviruses from bats. Phylogenetic trees A to G **(**Fig. 4) were constructed to assess the evolutionary relatedness of the Zimbabwean sequences to reference strains based on the regions identified in Fig. 3. In trees A, B, C, D, and G, the Zimbabwean sequences clustered closely with those from Uganda and Rwanda, while the Kenyan strain appeared to represent an earlier evolutionary stage compared to the other African strains (Fig. 4). In Fig. 3 the Kenyan genome shows great similarity in the conserved regions of the genome (Region A, B, C & G) but the similarity drops significantly in the spike region to around 80%. MAG1042 from the *Nycteris macrotis* bat displayed more recent evolutionary divergence across trees A to G compared to the *Rhinolophus simulator* bat strains in our Zimbabwean genomes.

In trees E and F, which represent the Spike (S) gene, Tree E showed the Zimbabwean genomic sequences clustering as older, more distant strains compared to other African sequences. However this tree was unsupported by bootstrap values, making it unreliable. In contrast, Tree F, supported by high bootstrap values, showed the *Nycteris macrotis*-MAG1042 branching earlier than those from Rwanda and Uganda. Zimbabwean *Rhinolophus simulator* sequences (MAG Cons) formed a branch within a sister clade of the African strains.

### Receptor-binding domain (RBD) analyses of the spike protein

**Fig. 5:**
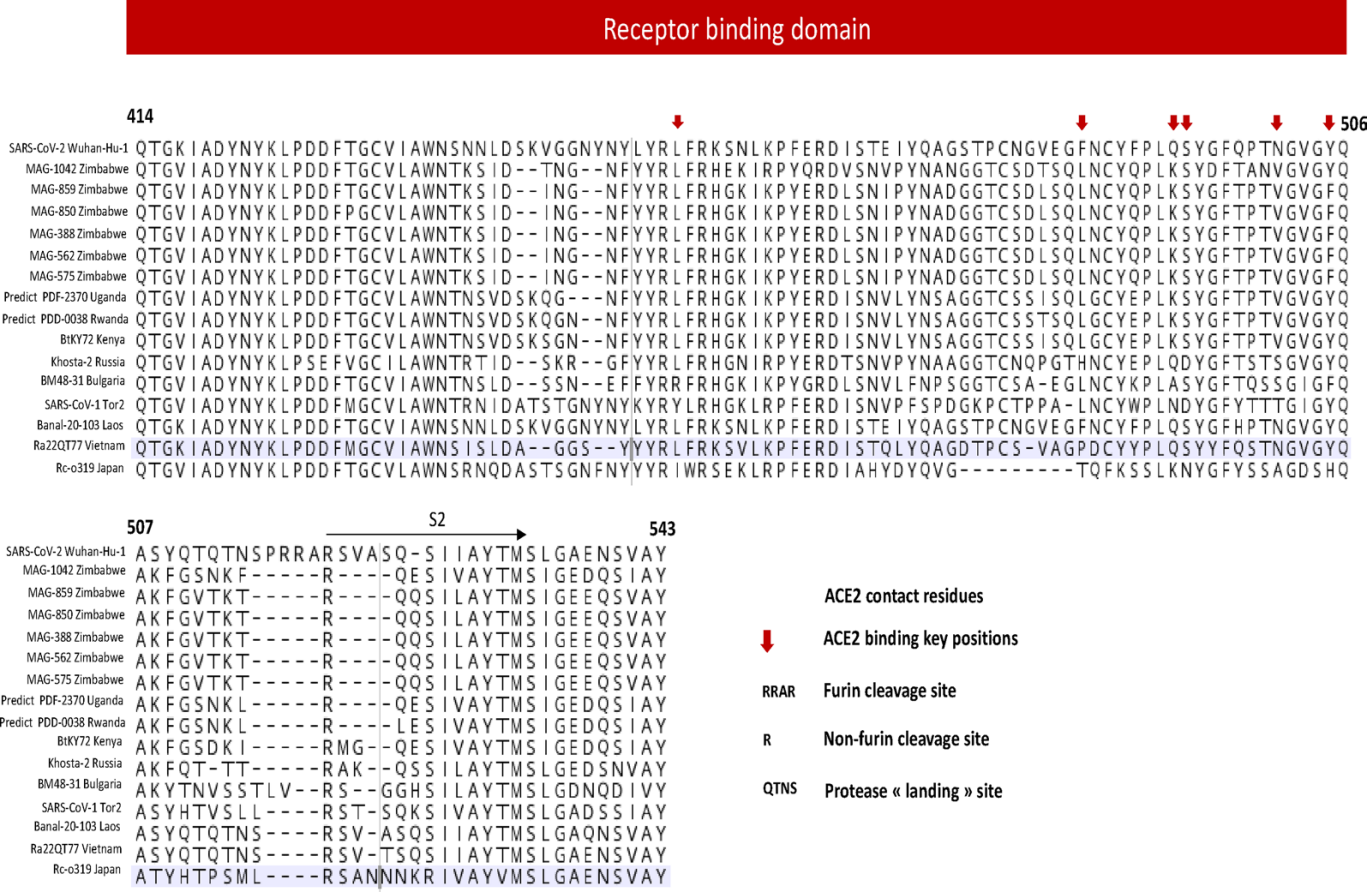
Amino acid alignment of the spike receptor-binding domain (RBD) region of Zimbabwean bat sarbecoviruses (highlighted in grey) compared to other African, European, and Asian *Sarbecovirus* from bats, including SARS-CoV-1 -Tor2 & SARS-CoV -2 (Wuhan-Hu-1). The alignment highlights amino acid conservation and variation within the RBD, particularly at key ACE2 contact residues (indicated by red arrows). Zimbabwean sequences show multiple substitutions at critical contact points and deletions in the receptor-binding motif (RBM), potentially impacting ACE2 receptor interaction

The figure below (Fig. 5) shows sequence alignment of the receptor-binding domain (RBD) of the spike protein bat sarbecoviruses characterised from Zimbabwean samples, Africa, Asia and Europe and human SARS-CoV-1 & -2. Zimbabwean genomic sequences exhibit multiple mutations at both ACE2 contact residues and binding key positions. Additionally they lack the furin cleavage site and show variations in protease cleavage motifs (landing sites) suggesting varied mechanisms of host cell entry.

All Zimbabwean bat sarbecoviruses RBD sequences showed amino acid substitutions at key ACE2-interacting residues, including K417V, G446N, G476N-*Nycteris* and G476D-*Rhinolophus*, F486L, Q493K, Q498T, and N501V, relative to SARS-CoV-2. Furthermore, the *Nycteris macrotis*- genome MAG1042 displayed additional amino acid differences compared to *Rhinolophus simulator* sequences, with unique substitutions at D476N, I471V, K462R, E465Q, L468V, G459E, G496D and T500N. At position 505, MAG1042 retained Y505, which is conserved in SARS-CoV-2, while the Rhinolophus sequences showed a Y505F substitution.

Additionally, we observed a receptor binding ridge in our sequences (amino acid positions 472–491) with positions of importance at 482–485. While SARS-CoV-2 had a GVEG motif, SARS-CoV-1 had PPA, Ugandan & Kenyan genomes had SISQ, and Rwandan genomes had STSQ in the same position, our sequences showed distinct variations: *Nycteris* MAG1042 contained DTSQ and *Rhinolophus* MAG consensus sequences had DLSQ motif.

## Discussion

Bats harbour numerous sarbecoviruses with great diversity influenced by long-term co-evolution [8, 9, 33]. The discovery of bat sarbecoviruses closely related to SARS-CoV-1 which emerged in 2003 and SARS-CoV-2 in 2019 emphasized the zoonotic potential of bat CoVs and their importance to public health [2, 4, 8, 9, 34]**.** Although extensive full genome surveillance has revealed diverse sarbecoviruses in Asian bats, particularly in *Rhinolophus* genus [8, 9, 34], our understanding of African bat sarbecoviruses remains limited [20]. To date, most identified bat sarbecoviruses are not known to infect humans directly. However, their genomic diversity and evolutionary dynamics highlight the need for continued surveillance.

A comprehensive analysis was conducted on the full bat sarbecoviruses genomes obtained from Zimbabwe and were compared with genetic references from bat species across Africa, Europe, and Asia, as well as SARS-CoV-1 & -2 sequences from humans. Phylogenetic analysis revealed that Asian bat sarbecoviruses displayed high genetic diversity and formed multiple distinct clusters. In contrast, fewer lineages have been described in Africa, within which our Zimbabwean sequences clustered closely. The greater observed diversity among Asian sequences is likely influenced by the significantly higher number of studies, extensive characterization efforts, and broader geographic sampling in Asia compared to Europe and Africa, as reflected in the publicly available genomic data.

Moreover, although African genomes clustered together, the notable branching differences observed in Fig. 2, particularly with the Kenyan full genome compared to other African sequences may suggest host-associated diversification among different Rhinolophus species across regions, similar to patterns seen in Asia. However, this hypothesis remains difficult to confirm due to limited host species identification and the low number of full genome sequences available from Africa.

Within the Zimbabwean sequences, bat sarbecoviruses full genomes characterised from *Rhinolophus simulator* bats MAG388, MAG562, MAG575, MAG850, and MAG859 samples shared a nucleotide identity of 99.8% (Table 2). We observed that *Nycteris macrotis*-MAG1042 demonstrated a high degree of genetic similarity (with 96.7% of nucleotide identity) to *Rhinolophus simulator*-MAG Cons sequences (MAG388, MAG562, MAG575, MAG850, and MAG859) across most genomic regions. This finding underscores the strong genetic coherence present in the genomes of bat sarbecoviruses from the same geographical site in Zimbabwe.

Notably, a divergence and a reduced percentage identity of 88% between MAG-1042 and MAG-Cons, hosted by different bat families, in the spike (S) protein was observed. This finding indicates that, despite the overall genetic coherence observed among the bat sarbecoviruses sequences of bats from the same geographical location, there is ongoing evolutionary change in the S region. Such divergence is likely driven by adaptive pressures, including host immune responses or modifications in receptor binding [35]. Additionally, this variation underscores site-specific mutations within different bat hosts, which may affect viral fitness and transmissibility specific to each bat species [35, 36]. This pattern highlights the critical need to monitor the spike region due to its significant role in host-virus interactions and potential for cross-species transmission.

Human SARS-CoVs and bat sarbecoviruses vary in three regions of the whole genome, the spike glycoprotein (S), ORF3 and ORF8 [36]. ORF8 is an accessory protein that facilitates immune evasion by generating early antibody responses [37–39], thus acting as a decoy while allowing viral entry. Furthermore, it reduces major histocompatibility complex (MHC) class I expression, allowing the virus to avoid detection by the host immune system [4]. A complete absence of ORF8 in Zimbabwean bat sarbecoviruses genomes was observed. This absence of Zimbabwean bat sarbecoviruses ORF8 raises critical concerns about viral evolution, host–pathogen relationships, and raises questions on zoonotic status of these viruses in potential spillover events. Furthermore, absence of ORF8 potentially increases the vulnerability of these viruses to immune clearance as the primary function of ORF8 is host immune evasion, highlighting a significant difference in pathogenic potential of bat sarbecoviruses and human-SARS-CoVs [40].

Eventually this observed absence of ORF8 in other African [19, 20] and European [21, 27] bat sarbecoviruses including the newly described Zimbabwean genomes may reflect an evolutionary state prior to the acquisition of OFR8 in Asian lineages given their basal phylogenetic position, however, this interpretation should be made cautiously as sampling bias given the limited genomic data from Africa and Europe may influence tree topology. While it remains uncertain whether ORF8 was never present in these lineages or subsequently lost, the current evidence favours true absence rather than deletion.

We suggest a comparative genomic approach as essential in clarifying patterns of ORF8 absence in bat sarbecoviruses, and identification of associations with other genomic changes which could impact transmission dynamics and pathogenicity. There is need for prioritization of monitoring understudied bat populations, particularly in places of high biodiversity, to prevent future spillover occurrences and expand our understanding of viral dynamics in bats.

Coronaviruses' envelope spike (S) protein facilitates virus entry into host cells by binding to the cellular receptor angiotensin-converting enzyme 2 (ACE2) in humans [41–43]. In this study, variations were identified in the ACE2 receptor binding domain (RBD) contact residues of Zimbabwean bat sarbecoviruses genomes compared to the Wuhan human SARS-CoV-2 genome, a pattern consistent with findings from other African bat sarbecoviruses variants reported in Uganda, Kenya, and Rwanda, as thoroughly examined by Wells et al.,2021 [15]. These RBD variations of Zimbabwean sarbecovirus suggest a low affinity for human ACE2 in the insectivorous bats, implying that humans are less likely to be directly infected by these viruses.

Notably, the amino acid substitutions at positions K417V (Lysine/Valine), G446N (Glycine/ Asparagine), G476N (Glycine/Asparagine), F486L ( Phenylalanine/Leucine), Q493K (Glutamine/Lysine), Q498T (Glutamine/Threonine), and N501V (Asparagine/ Valine) in the Zimbabwean sequences indicate a low binding affinity of bat sarbecoviruses to human ACE2. These residues are key determinants of ACE2 interaction, influencing viral infectivity and host range [44, 45]. N501 is known to interact with the K353 hotspot on human ACE2 [15]; however, in our sequences, it is substituted by valine (N501V), introducing a hydrophobic residue that likely weakens this interaction. This substitution, as reported by Wells et al. (2021), disrupts the favorable contact with K353, thereby lowering binding affinity to human ACE2 [15]. Thus, the identified substitutions are consistent with a diminished ability of these bat sarbecoviruses to bind human ACE2, thereby lowering the likelihood of direct zoonotic transmission. However these substitutions may reflect the adaptation of the virus to other animal species and/or bat populations [46, 47].

Furthermore, we observed the presence of the receptor-binding ridge in our sequences (Fig. 5). Wells et al. (2021) reported that this region is absent in all non-ACE2 binders [15], suggesting that African bat sarbecoviruses, including our Zimbabwean sequences, are likely ACE2 users, primarily in bats as they show low affinity for human ACE2. Although the ridge is present, the key GVEG motif in SARS-CoV-2, which enhances binding to human ACE2 by forming a compact loop [48], was replaced in our sequences. Specifically, *Nycteris macrotis* MAG1042 had a DTSQ motif, while *Rhinolophus* MAG Cons sequences contained DLSQ. Wells et al. also reported SISQ and STSQ motifs in other African sarbecoviruses and concluded these likely reduce human ACE2 binding [15].

Furthermore, the absence of furin cleavage sites in the bat SARSr-CoV sequences including those from Zimbabwe underscores the low transmissibility and pathogenicity of these viruses in humans. However, the lack of this site does not entirely eliminate the possibility of human infection. Notably, the presence of proteins at non-furin cleavage sites suggests that bat sarbecoviruses may utilize proteases different from those of human SARS-CoVs for viral entry. This alternative mechanism could play a crucial role in the virus ability to cross species barriers. Variations were also identified in the protease landing sites of the Zimbabwean bat SARSr-CoV sequences compared to human SARS-CoV-2 and Chinese bat sarbecoviruses. These differences suggest that African, European, and other Asian bat sarbecoviruses may require distinct host conditions and proteases for efficient entry into host cells, in contrast to human SARS-CoV-2.

Genetic variations were also observed between genomes of the two bat families in Zimbabwe. ACE2 contact residues of *Nycteris macrotis*-MAG1042 differed from the other *Rhinolophus simulator*-Zimbabwean sequences. MAG1042 displayed unique substitutions at positions K462R, G459E, E465Q, L468V, D470N, I471V, and T500N. Notably, MAG1042 retained a tyrosine (Y505), which is conserved in SARS-CoV-2, while the *Rhinolophus* sequences exhibited a Y505F substitution. The G496D mutation involves a shift from a small, non-polar glycine to a negatively charged aspartic acid, leading to loss of charge and polarity. The T500N substitution introduces changes in hydrogen bonding potential and steric configuration. These modifications reduce the binding affinity of MAG1042's spike protein to human ACE2, thereby diminishing the virus’ potential for direct human infection. In other studies, Tyrosine at position 505 has been associated with stronger ACE2 interactions in both human and certain animal hosts [49, 50], however in our case it is the opposite. These amino acid changes are predicted to reduce the affinity of the MAG1042 spike protein for human ACE2, thereby decreasing the likelihood of direct human infection.

This suggests that, while bat sarbecoviruses from Zimbabwean bats are less adapted to humans, RBD polymorphism could have a substantial impact on the virus's host range and cross-species transmission among bat species [16, 35]. The ability of CoVs to adapt to different hosts through amino acid residue alterations in the RBD is crucial for enabling cross-species infections [48]. Multiple mutations in the RBD were detected in the Zimbabwean sequences, showing significant divergence from those identified in SARS-CoV-2 and other bat sarbecoviruses. These changes may reflect ongoing evolutionary adaptations, potentially influencing the virus ability to interact with ACE2 receptors from different hosts, thereby expanding its host range and cross-species transmission [48, 51].

## Conclusions

The complete absence of ORF8, coupled with the lack of both furin and non-furin cleavage sites, suggests that the bat sarbecoviruses found in Zimbabwe exhibit reduced virulence and zoonotic potential compared to human-infecting SARS-CoVs such as SARS-CoV-1 &-2. Despite these differences, the importance of further investigation into other viral components that may compensate for these missing features remains critical. Although these viral strains may pose a lower immediate risk to human health, ongoing surveillance of bat coronaviruses is necessary to gain a comprehensive understanding of their evolutionary dynamics and the potential emergence of future.

## Supplementary Information

Below is the link to the electronic supplementary material.

Supplementary Material 1

## Data Availability

All sequences were deposited in GenBank under accession numbers: PV536206, PV536207, P536208, PV536209, PV536210, PV584046 https://www.ncbi.nlm.nih.gov/nuccore/?term=PV536206:PV536210+OR+PV584046.
