# Supplementary material for: Complete genome sequence analysis of Sarbecovirus (severe acute respiratory syndrome-related coronaviruses) from Zimbabwean insectivorous bats
Source: BMC Genomics. 2025 Sep 26;26:828. doi: 10.1186/s12864-025-12015-9 (PMC12465345; doi:10.1186/s12864-025-12015-9)
Supplement: Supplementary file 1 — Supplementary Material 1 [file 12864_2025_12015_MOESM1_ESM.docx]

**Additional files**

**Additional file 1**: Primers used for the Zimbabwean Bat SARSr-CoV full genomes amplification

**Additional file 2**: Scheme of Zimbabwean Bat SARSr-CoV full genomes strategy

**Additional file 3**: Reference sequences used in this study
